# Dosimetry for Small Fields in Stereotactic Radiosurgery Using Gafchromic MD-V2-55 Film, TLD-100 and Alanine Dosimeters

**DOI:** 10.1371/journal.pone.0063418

**Published:** 2013-05-09

**Authors:** Guerda Massillon-JL, Diego Cueva-Prócel, Porfirio Díaz-Aguirre, Miguel Rodríguez-Ponce, Flor Herrera-Martínez

**Affiliations:** 1 Instituto de Física, Universidad Nacional Autónoma de México, D.F., Mexico; 2 Conclina C.A. Hospital Metropolitano, Quito, Ecuador; 3 Hospital San Javier, Guadalajara, Mexico; 4 Instituto Nacional de Cancerología, Mexico, D.F., Mexico; Dresden University of Technology, Germany

## Abstract

This work investigated the suitability of passive dosimeters for reference dosimetry in small fields with acceptable accuracy. Absorbed dose to water rate was determined in nine small radiation fields with diameters between 4 and 35 mm in a Leksell Gamma Knife (LGK) and a modified linear accelerator (linac) for stereotactic radiosurgery treatments. Measurements were made using Gafchromic film (MD-V2-55), alanine and thermoluminescent (TLD-100) dosimeters and compared with conventional dosimetry systems. Detectors were calibrated in terms of absorbed dose to water in ^60^Co gamma-ray and 6 MV x-ray reference (10×10 cm^2^) fields using an ionization chamber calibrated at a standards laboratory. Absorbed dose to water rate computed with MD-V2-55 was higher than that obtained with the others dosimeters, possibly due to a smaller volume averaging effect. Ratio between the dose-rates determined with each dosimeter and those obtained with the film was evaluated for both treatment modalities. For the LGK, the ratio decreased as the dosimeter size increased and remained constant for collimator diameters larger than 8 mm. The same behaviour was observed for the linac and the ratio increased with field size, independent of the dosimeter used. These behaviours could be explained as an averaging volume effect due to dose gradient and lack of electronic equilibrium. Evaluation of the output factors for the LGK collimators indicated that, even when agreement was observed between Monte Carlo simulation and measurements with different dosimeters, this does not warrant that the absorbed dose to water rate in the field was properly known and thus, investigation of the reference dosimetry should be an important issue. These results indicated that alanine dosimeter provides a high degree of accuracy but cannot be used in fields smaller than 20 mm diameter. Gafchromic film can be considered as a suitable methodology for reference dosimetry. TLD dosimeters are not appropriate in fields smaller than 10 mm diameters.

## Introduction

Conventionally, reference dosimetry in small stereotactic radiosurgery (SRS) radiation fields using linear accelerator (linac) modality treatments is performed by measuring the absorbed dose to water rate in a large reference radiation field of 10×10 cm^2^ and using an output factor that accounts for the differences between the conventional reference field and the small field of interest (of the order of a few mm^2^). Existing published protocols such as IAEA-TRS-398 and AAPM-TG-51 [1]–[2] provide a method and radiological parameters needed for the measurement of the absorbed dose to water rate at a reference 10×10 cm^2^ field using ionization chambers, whereas the output factors are measured and/or calculated by the users, [3]–[7]. Contrary to the linac, in some specific machines such as in a Leksell Gamma Knife (LGK) unit, the large reference 10×10 cm^2^ field size required by the existing protocols even does not exist. In these fields, the determination of absorbed dose to water rate to the different nominal radiation fields is frequently performed by calculating the product of the absorbed dose to water rate measured in the largest available radiation field (generally 16 mm or 18 mm diameter) using a small volume ion chamber according to the conventional protocols and the output factor provided by the vendor (or user measured) for each nominal field. In this work, conventional dosimetry (CD) is referred to the absorbed dose to water rate determined with ionization chamber following IAEA-TRS-398 or AAPM-TG-51 [1]–[2] protocols for the linear accelerator and AAPM-TG-21 [8] for the Leksell Gamma Knife unit and the use of output factors to account for the differences between the reference field and the small field.

After many years of investigations, the major concerns for reference dosimetry in small radiation fields used in stereotactic radiosurgery still remain. This is due to the difficulty to perform accurate dose measurements caused by various issues such as: partial occlusion of the direct beam photon source’s view from the measurement point [6], lack of lateral charged particle equilibrium, steep dose-rate gradient, volume averaging effect on the detector response and variation of the energy fluence in the lateral direction of the beam [6], [9]–[10]. Due to these difficulties, the ideal dosimeters to be used in these radiation fields must be not only water-equivalent, but also have a small size and the possibility to provide sub-millimeter resolution [9]. Ionization chambers that are commonly used in CD fields are not suitable in small and nonstandard radiation fields because of a lack of spatial resolution and accuracy in the absorbed dose measurements caused by the fluence perturbation. Besides this, a recent study that quantified perturbation factors for small ionization chambers in small field dosimetry has revealed that even 0.016 cm^3^ volume ionization chambers are not suitable to be used in a 0.8×0.8 cm^2^ field [11]. Another disadvantage of the small ionization chamber is the amount of charge collected within the radiation field which can be comparable to the leakage of the dosimetry system itself. Regarding the output factors, it has been reported that for very small radiation fields (≤ 20 mm diameter) and under similar conditions, the output factors estimated as a ratio of detector reading can vary between 12% and 14%, depending on the detector used and the institution where the experiment was performed [6], [12]. Such a remarkable difference could possibly be associated to a volume averaging effect [10] and/or the absence of water equivalence of the detectors used [9].

To respond to this problem, an international collaboration [10] proposed the use of an intermediate reference field, *f_msr_*, which denotes a machine-specific reference field, for static modalities or treatment machines such as the Gamma Knife and radiosurgical collimators that cannot establish a conventional reference field. In this case, the absorbed dose to water rate 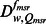 at the reference depth in a beam quality *Q_msr_*_,_ reference field *f_msr_*_,_ and in the absence of the chamber is given by [10]:
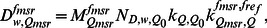
(1)where 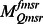 is the corrected reading of the dosimeter in the field *f_msr_*, 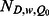 is the calibration coefficient in terms of absorbed dose to water, *w*, for an ionization chamber in a reference beam quality *Q*_0,_ *k_Q_*_,*Q*0_ is the beam-quality correction factor, which corrects for the differences between the reference beam quality *Q_o_* at the standards laboratory and the beam quality *Q* of the conventional reference field *f*_ref_, and 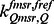 is a correction factor that accounts for the difference between the responses of an ionization chamber in the fields *f*_ref_ and *f*_msr_ defined by the following equation [10]:



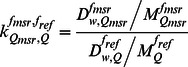
(2)From a statistical point of view, the extension of the established code of practice by using an additional correction factor 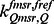 for the absorbed dose determination in the machine-specific reference field would result in a less accurate absorbed dose to water rate measurement.

Until now, most of the data reported in the literature about dosimetry in small fields of stereotactic radiosurgery are related to relative dosimetry such as measurements of output factors, tissue maximum ratios, among others [12]–[14]. However, not much attention has been given to the reference dosimetry such as in a Gamma Knife unit where the dosimeter used to calibrate the reference field could be an important topic. Recently, it was reported that, if a strict and careful experimental procedure is followed, Gafchromic films and thermoluminescent dosimeters (TLD) can be used to calibrate directly small radiation fields in terms of absorbed dose to water rate with a relative standard uncertainty of 3 to 4% [15]–[16]. Furthermore, it has been considered important to identify and evaluate new dosimeters that are appropriate for reference dose measurements in small and non-standard radiation fields due to the unsuitability of commercially available ionization chambers used for measurements in high dose gradient radiation fields [17]. Novotny and collaborators [17] have investigated the accuracy of alanine to be used as reference dosimeter for small LGK fields by comparing the absorbed dose measured with an A16 Exradin ionization chamber (0.007 cm^3^ volume) and that from alanine pellets (4.8 mm diameter, 2.0 mm height) irradiated at the center of a spherical polystyrene phantom using a 16 mm and 18 mm collimator Perfexion and 4C LGK unit, respectively. Depending on the absorbed dose value, differences up to 1.7% and 1.2% were reported between the two methods for 16 mm and 18 mm collimators, respectively [17].

In this work, the absorbed dose to water rate for 4 Leksell Gamma Knife collimators (4 mm to 18 mm) and 5 radiation field diameters of a SRS modified linear accelerator (adapted cylindrical collimators from 7.5 mm to 35 mm diameters) was evaluated directly in water using thermoluminescent dosimeters (TLD-100), Gafchromic films (MD-V2-55) and alanine pellets to investigate the suitability of reference dosimetry measurements with good accuracy (better than 1.5%) for small radiation fields using calibrated passive dosimeters. In particular, the absorbed dose to water rate obtained with each dosimeter in the small fields was compared to that computed with ionization chambers following the conventional dosimetry protocols according to the modality treatment as described above.

## Materials and Methods

### Calibration of Reference Beams

The reference 10×10 cm^2^ fields in an 6 MV x-ray accelerator Elekta Synergy and a Theratron 1000 ^60^Co gamma beam were calibrated in terms of absorbed dose to water following the IAEA TRS-398 protocol [1]. Measurements were performed using a source surface distance (SSD) of 95 cm at 5 cm depth in water phantoms model PTW-MP3 for the ^60^Co gamma beam and Welhöfer Scanditronx IBA for the Elekta Synergy. The reference dosimetry system used in this work consisted of a 0.6 cm^3^ Farmer type ionization chamber model PTW23333 and an electrometer model Standard Imaging CDX-2000A calibrated at the National Institute of Standards and Technology (NIST). The reference dosimetry system was used simultaneously with a) a 0.6 cm^3^ FC65P Welhöfer Scanditronix ionization chamber associated with a Dosimetry Dose1 IBA electrometer calibrated at the University of Wisconsin, USA, for the Elekta Synergy accelerator; and b) a 0.125 cm^3^ semiflex PTW31010 chamber with a Freiburg PTW T10001-11509 electrometer calibrated at PTW, Germany, for the Theratron 1000 ^60^Co beam. Temperature and pressure were monitored during the measurements by digital thermometer Fluke 1523 and Druck DPI 12 barometer.

### Dosimeter Preparation and Readout

The dosimeters used included TLD-100 chips with dimensions 3.1×3.1×0.89 mm^3^ (all belonging to a single batch); Gafchromic film model MD-V2-55 with batch number Q0304MDV2 and alanine pellets, 4.9 mm diameter and 3.0 mm height, belonging to batch number T030901. Following a protocol previously reported [18], the TLDs annealing procedure was performed in air at a temperature of 400°C for 1 h, cooled down during 30 min and followed immediately by a second anneal at 100°C for 2 h. The TLDs were read 48 h after the irradiation using a TLD reader model Harshaw 3500 at a heating rate of 8°C/s, integrating from room temperature to 400°C. In this work, the TL signal is defined as the net integral of the glow curve after subtraction of background (signal not due to the irradiation) and glow peak 2 [18]. For the Gafchromic film, 1×1 cm^2^ pieces were cut and read 48 h before irradiation. After the irradiation, each piece was read with a HP Scanjet 7650 document flatbed scanner using the transmission mode with 48 bit-RGB color depth and a spatial resolution of 300 dpi (85 µm) according to the established protocol in our laboratory [19]. The image analysis of the film was made using the ImageJ public software [20] by selecting a region of interest (ROI) equal to 5 mm and 2 mm diameter in the calibration and the small fields, respectively. The three colour channels from the film image were extracted and considering the high dose rate in the radiation fields, the green channel was used to evaluate the absorbed dose in both conditions. Alanine pellets were provided by NIST in the USA and mailed to Mexico City to calibrate the Theratron 1000 ^60^Co gamma beam in terms of absorbed dose to water. The alanine dosimeters were irradiated under conditions similar to the reference dosimetry system in the Theratron 1000 ^60^Co gamma beam to several doses and returned to NIST to be read out according to the NIST reading protocol [21]. For each irradiated alanine dosimeter, the EPR signal with its associated absorbed dose value from the ^60^Co gamma irradiation was provided, in addition to the NIST calibration curve for this batch.

### Calibration of the Dosimeters

Gafchromic film, TLDs and alanine dosimeters were calibrated in terms of absorbed dose to water in the reference Theratron 1000 ^60^Co gamma-ray and Elekta Synergy 6 MV x-ray beams, using the dose rate determined with our reference dosimetric system. Three TLDs per each dose value were exposed to dose levels ranging from 40 mGy up to 1 Gy. For the Gafchromic film, four pieces per absorbed dose value were irradiated at doses between 0.5 Gy and 75 Gy, while two alanine dosimeters per dose value were utilized for the dose interval between 24 Gy and 50 Gy.

**Figure 1:**
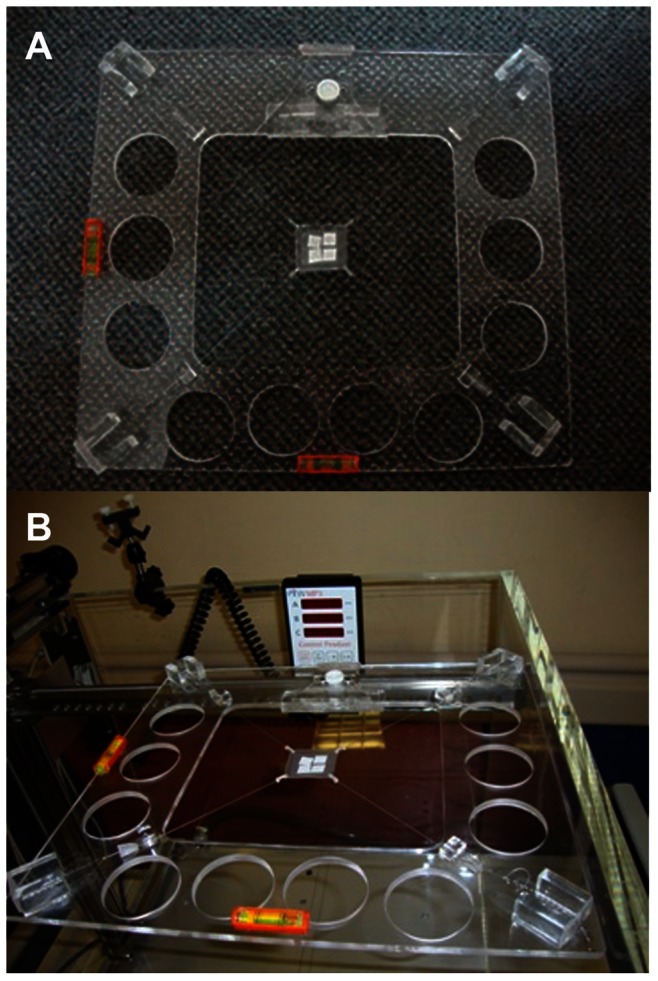
Figure 1. Polymethyl methracrylate holder. a. With a sealed bag. b. Mounted in the water phantom.

To irradiate the dosimeters directly in the water phantom, a spring loaded jig of 35×35×0.5 cm^3^ (Figure 1a) was designed and built as a holder at Hospital Metropolitano, Quito, Ecuador. The holder was made of polymethyl methracrylate (PMMA) and consisted of an empty area of 20×18 cm^2^ at the centre for the passage of the radiation beam and 10 circular holes of 5 cm diameter in the sides to allow the water to flow freely and avoid any perturbation in the radiation field. Two bubble level tools were also positioned over the holder in the x and y directions to level the dosimeters in the water phantom. Water-proof packages of 5×5 cm^2^ were prepared to place the dosimeters. After insertion of the dosimeters, the packages were vacuum sealed using a commercially available food saver device and punched in each corner in order to be mounted in the spring loaded jig that supported them in the water phantom at 5 cm depth perpendicular to the radiation beam. Figure 1b displays the spring loaded jig mounted inside the water phantom with a sealed package. After irradiations, the TLDs as well as the Gafchromic film were read at Instituto de Física, UNAM, Mexico, while the alanine dosimeters were read at NIST. To validate the absorbed dose to water rates obtained in the vacuum sealed package, a comparison was done between a NIST calibration curve from ^60^Co gamma-rays for this alanine dosimeter batch and our measurement with ionization chamber.

### Irradiations in Small Radiation Fields

For the measurements in the small SRS radiation fields, all three detectors previously calibrated and mentioned above were used. The absorbed dose to water rate in the small radiation fields was evaluated in a Clinac 600 modified for stereotactic radiosurgery at the same SSD and depth as in the reference field. For the Gamma Knife unit, the dosimeters were positioned at the centre of a spherical solid plastic phantom made of acrylonitrile butadiene styrene (ABS plastic) at 40 cm source-detector distance (SDD). In this case, the plastic phantom has specific holes to position an ionization chamber, the TLD and alanine dosimeters according to their size at the unit focus point (LGK mechanical centre where all the 201 ^60^Co beams intersect). The absorbed dose to water rates were computed for 4 mm, 8 mm, 14 mm and 18 mm helmet in the Gamma Knife unit, and 5 collimator sizes (7.5, 10, 15, 25, 35 mm diameter) for SRS with modified linear accelerator. Due to the small size of these radiation fields, each dosimeter was irradiated individually. Thus, under this irradiation condition, any variation in the reproducibly of the dosimeter position, would be reflected in the standard deviation of the average response among the different dosimeters. For each exposure, the delivered dose was around 0.5 Gy, 25 Gy and 35 Gy for TLDs, Gafchromic film and alanine, respectively. The selection of these absorbed dose values was based on the independence of these dosemeters on absorbed dose-rate [18]–[19] and on the evaluation of the combined standard uncertainties as a function of dose reported previously for each dosimeter. For example, relative combined standard uncertainty of less than 1.5% in the absorbed dose determined with MD-V2-55 film irradiated at 25 Gy with high energy photons for the green colour channel has been reported following our protocol [19], while for TLD-100 irradiated at 0.5 Gy, combined standard uncertainty of 3%–4% was reported [18].

### Uncertainty Evaluation

The uncertainties in the absorbed dose to water rate in each radiation field (reference and small radiotherapy fields) were evaluated following the standard procedure of combining uncertainties described elsewhere [22]–[24] considering a 68% confidence limit, i.e. a coverage factor of *k = 1*. The combined standard uncertainty of the absorbed dose to water rate was evaluated by adding quadratically the standard uncertainty in the ionization chamber’s collected charge, calibration factor, thermometer and barometer reading, time exposure and position distance for the reference field. For the small fields, the standard uncertainty in the dosimeter’s response, calibration curve of each dosimeter and total exposure time were considered.

## Results

### Calibration of the Reference Radiation Fields

**Figure 2:**
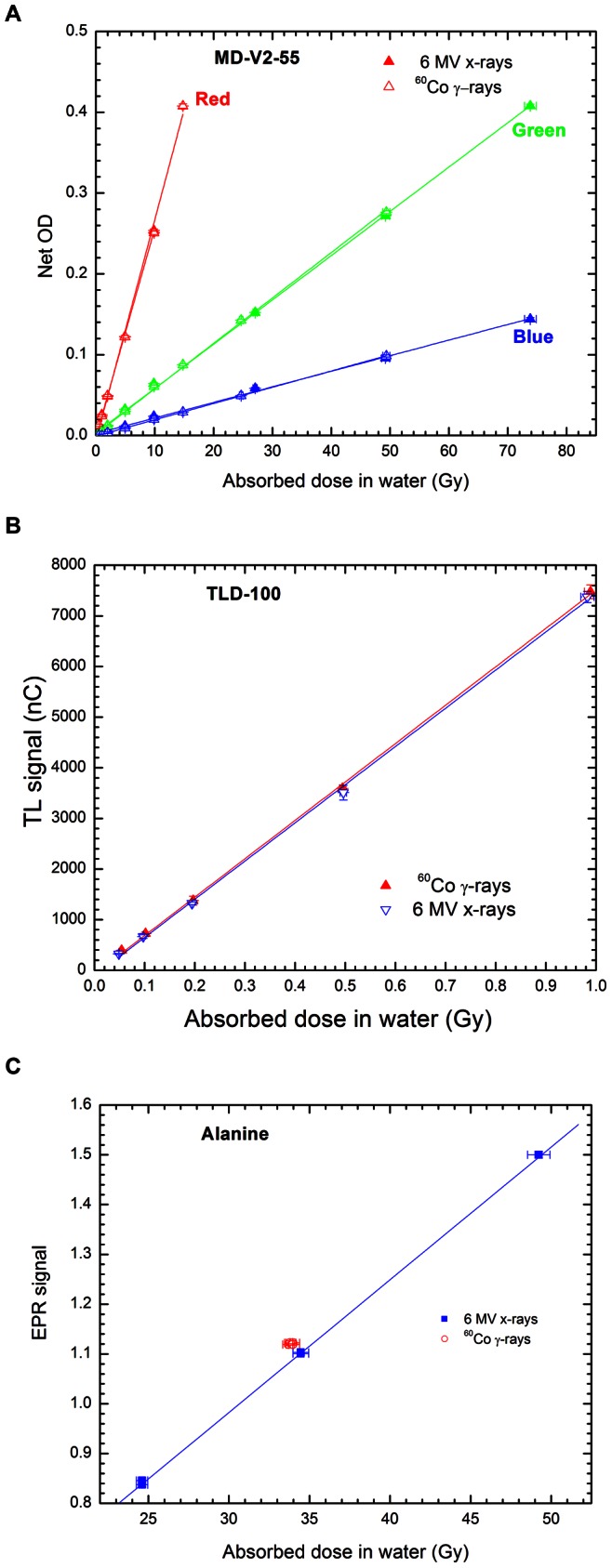
Figure 2. Calibration curves for each dosimeter exposed to 60Co gamma and 6 MV x-ray beams as a function of the absorbed dose to water. The symbols represent the data and the lines are linear fits: a. MD-V2-55 for the three color channels (red, green and blue). b. TLD-100. c. Alanine dosimeters and for the 60Co data, we only compare the dose determined with our ionization chamber and that provided by NIST.

The absorbed dose to water rate determined in the reference 10×10 cm^2^ radiation fields with the different ionization chambers compared to that obtained with the alanine pellet inside the water-proof package are shown in Table 1. Good agreement within 0.04%–1.1% (relative combined standard uncertainties between 1.25%–1.63%, *k = 1*) is observed between the absorbed dose to water rate computed with the different chambers. Furthermore, the absorbed dose reported by NIST divided by the exposure time for the alanine dosimeter irradiated inside the water proof package statistically agrees within 0.65% with the absorbed dose to water rate evaluated directly in the water phantom with our ionization chamber. This indicates that the water proof package does not perturb the radiation fields and the attenuation of the beam in the material is negligible. Thus, all the measurements can be considered to be in water. Figures 2a, 2b and 2c display the calibration curves for Gafchromic film MD-V2-55 (the three colour channels), TLD-100, and alanine dosimeter, respectively after exposure to ^60^Co gamma and 6 MV x-rays in the water phantom. The data points represent the experimental measurements and the lines are linear fits. The netOD for the green channel (important for this study because of the lower uncertainty provided on the absorbed dose measurement), the TL and EPR signal as a function of the absorbed dose are described by the following equations for both energies:

**Table 1:** Absorbed dose to water rate determined in the 10×10 cm^2^ reference fields, SSD = 95 cm at 5 cm depth for 6 MV x-rays and ^60^Co gamma rays following TRS-398 [1].

Detectors	^60^Co gamma rays		6 MV X-rays	
	× 10^−3^ Gys^−1^	IC_1_/other	× 10^−3^ Gys^−1^	IC_1_/other
aIC_1_	11.292±0.141	1.000	9.733±0.131	1.000
bIC_2_	11.297±0.184	0.9995		
Alanine	11.220±0.083	1.0065		
cIC_3_			9.625±0.135	1.0112

a PTW (0.6 cm^3^) Farmer type 23333 calibrated at NIST, USA,

b PTW (0.125 cm^3^) Semiflex type 31010 calibrated at PTW, Germany,

c Scanditronix (0.6 cm^3^) type FC65P calibrated at University of Wisconsin, USA.

Gafchromic film MD-V2-55 (correlation coefficient *R* = 0.99975).



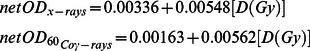
(3)TLD-100 (correlation coefficient *R* = 0.9998).
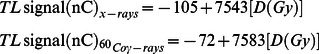
(4)


Alanine (correlation coefficient *R* = 0.9999).
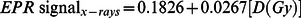
(5)


These equations were used to evaluate the absorbed dose in the small radiation fields: 6 MV x-rays for the modified linear accelerator and ^60^Co gamma for the Gamma Knife unit. For the alanine irradiated with ^60^Co gamma rays, the NIST calibration curve was used. Thus, the obtained value was divided by the exposure time or the monitor units (MU) to get the absorbed dose to water rate.

### Absorbed Dose to Water Rate in the SRS Field

**Figure 3:**
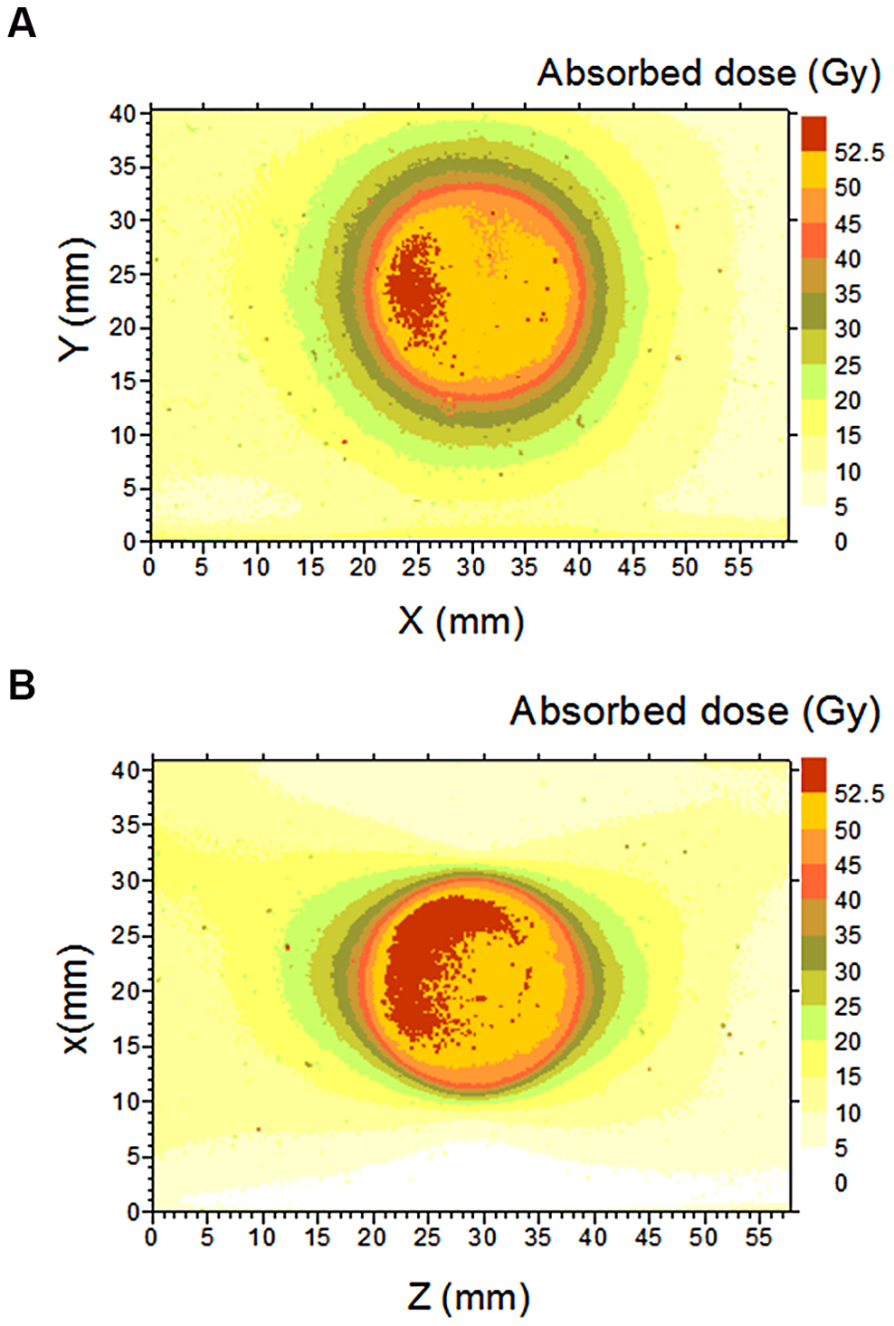
Figure 3. 2D dose distributions in the 18 mm diameter LGK field. a. Axial plane relative to the patient position. b. Coronal plane relative to the patient position.

**Figure 4:**
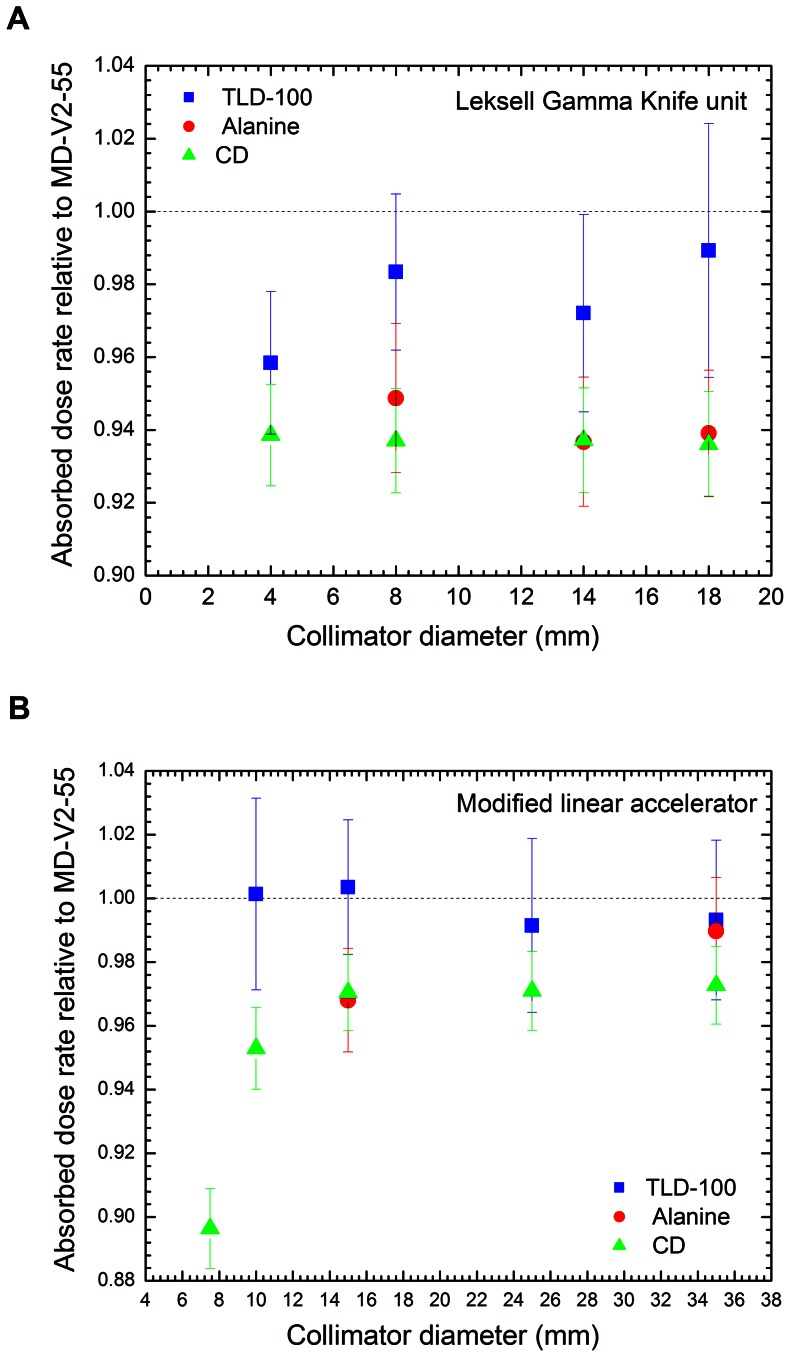
Figure 4. Ratio of the absorbed dose to water rate determined with each detector and that obtained with the MD-V2-55 film. a. Leksell Gamma Knife unit. b. Modified linear accelerator.

Tables 2 and 3 display the reference absorbed dose to water rate determined with Gafchromic film, TLD and alanine for the small radiation fields in the Leksell Gamma knife unit and the modified linear accelerator, respectively, compared with that evaluated with ionization chamber according to the conventional dosimetry (CD) protocols. As displayed in Table 2, for the Gamma knife unit and within measurement uncertainties, the difference between our data and that obtained through the CD protocol apparently depends on the dosimeter sizes, regardlesss the LGK collimators. This behaviour can be possibly due to a dosimeter volume averaging effect caused by the existence of absorbed dose gradient in the radiation field as shown in Figures 3a and 3b that displayed the 2D absorbed dose for the LGK 18 mm diameter collimator. For the modified linear accelerator; this difference is a function of the collimator diameter, independent of the dosimeter used. To better visualize this feature, the absorbed dose to water rate computed with each dosimeter was normalized to that obtained with MD-V2-55 film for both treatment modalities and depicted in Figures 4a and 4b.

**Table 2:** Reference absorbed dose to water rate computed in the Leksell Gamma Knife® unit compared with CD following the AAPM-TG21 [8].

		Collimator diameters (mm)			
		4	8	14	18
Dosimeter	Size	(mGy s^−1^)	(mGy s^−1^)	(mGy s^−1^)	(mGy s^−1^)
MD-V2-55	∼240^a^	20.18±0.30	22.23±0.34	22.92±0.35	23.31±0.36
TLD-100	3.1×3.1×0.89^b^	19.34±0.27	21.86±0.72	22.28±0.52	23.06±0.73
Alanine	4.9^c^×3.0^d^		21.09±0.32	21.47±0.24	21.89±0.22
CD		18.94	20.83	21.48	21.82

a µm thickness,

b mm^3^,

c mm diameter,

d mm thickness.

**Table 3:** Reference absorbed dose to water rate computed in the modified accelerator for SRS compared with CD following TRS-398 [1].

		Collimator diameters (mm)				
		7.5	10	15	25	35
Dosimeter	Size	(mGy MU^−1^)	(mGy MU^−1^)	(mGyMU^−1^)	(mGyMU^−1^)	(mGyMU^−1^)
MD-V2-55	∼240^a^	7.14±0.10	7.43±0.10	8.13±0.10	8.60±0.11	8.79±0.11
TLD-100	3.1×3.1×0.89^b^		7.44±0.20	8.16±0.14	8.53±0.21	8.73±0.19
Alanine	4.9^c^×3.0^d^			7.87±0.09		8.7±0.1
CD		6.4	7.08	7.89	8.35	8.55

a µm thickness,

b mm^3^,

c mm diameter,

d mm thickness.

### Absorbed Dose to Water Rate Relative Combined Standard Uncertainty in the Small Radiation Fields

**Figure 5:**
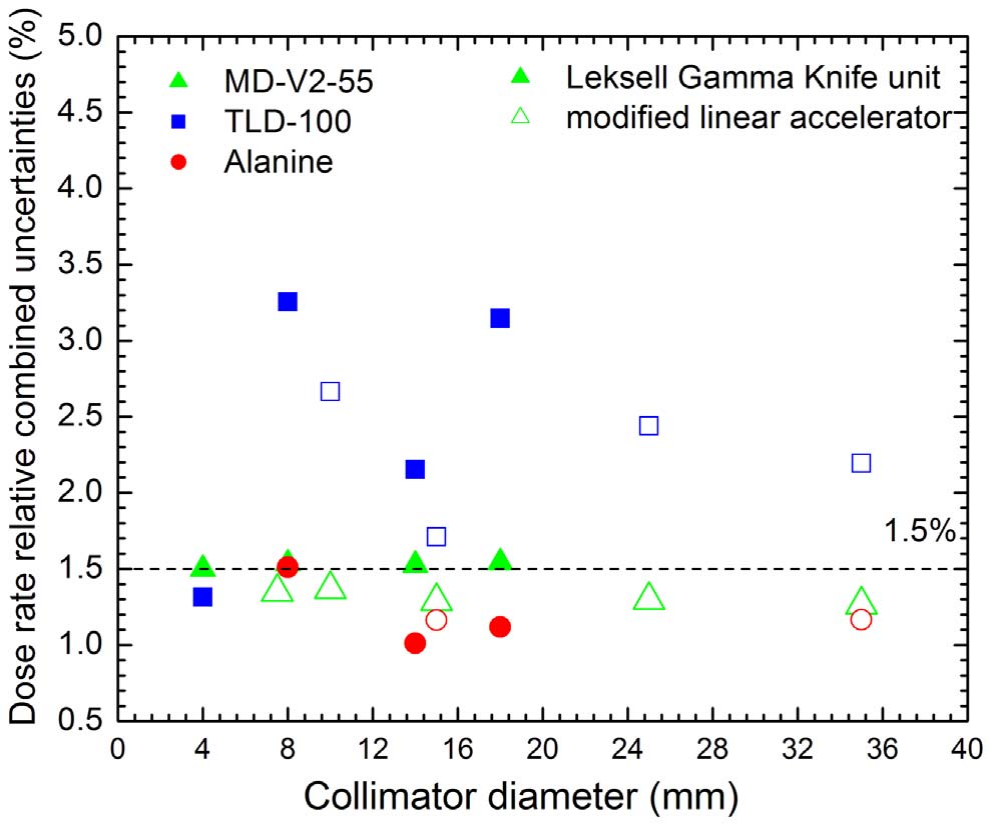
Relative combined standard uncertainties computed in this work. Full symbols are data for the LGK unit and open symbols represent data for the linac.

The relative combined standard uncertainties in the absorbed dose to water rate quantified for each small radiation field by the different dosimeters are presented in Figure 5. Note that, for all radiation fields, the relative combined standard uncertainty in the absorbed dose to water rate evaluated with the Gafchromic film and the alanine dosimeter is less than 1.5% (*k = 1*), while with TLD-100 uncertainties of up to 3.5% (*k = 1*) were obtained.

## Discussion

Reference absorbed dose to water rates for 4 Leksell Gamma Knife collimators and 5 radiation field diameters of a SRS modified linear accelerator were investigated using TLD-100, Gafchromic MD-V2-55 and alanine dosimeters. The reference 10×10 cm^2^ ^60^Co gamma and 6 MV x-ray radiation fields were calibrated with 3 ionization chambers, finding agreement of 0.04% to 1.1%, within measurement uncertainties.

According to data displayed in Table 2 and Figure 4a, the mean absorbed dose to water rate determined with alanine dosimeters and ionization chamber following the CD protocols relative to that obtained with MD-V2-55 film are, on the average, (0.941±0.011) and (0.937±0.007), respectively, regardless the LGK collimator diameter. By using TLD-100, the mean ratio is (0.981±0.015) for radiation fields larger than or equal to 8 mm diameter and (0.958±0.019) for 4 mm diameter. Note that this ratio systematically decreases as the dosimeter size increases and is statistically constant for collimator diameters larger than 8 mm. The dependence with the dosimeter size could be explained as a consequence of averaging volume effect caused by the dosimeter dimension (0.125 cm^3^ ionization chamber: ∼7.25 mm length and 6.9 mm diameter; alanine: 4.9 mm diameter and 3.0 mm thickness; TLD-100: 3.1×3.1×0.89 mm^3^; Gafchromic film: ∼240 µm thickness).

On the other hand, it is also interesting to note that, if the data obtained in this work from the Gamma Knife unit is used to evaluate the output factors (absorbed dose ratio between a small field and the 18 mm diameter field) as conventionally done, the result shown in Table 4 for the Gafchromic film and the alanine dosimeter is in agreement within 0.1%–0.8% with that obtained through CD protocols, except for TLD-100 where a difference of more than 3% is observed for the 4 mm diameter field. Comparing with data obtained for the Gafchromic film, the under-response in the 4 mm collimator diameter for TLD-100 can presumably be related to a lack of spatial resolution and/or an averaging volume effect caused by the TLD-100 dimensions (3.1 mm side vs 4 mm diameter field). Nevertheless, from the standpoint of reference absorbed dose displayed in Table 2, there is a sub-estimation of more than 6% depending of the dosimeter size. As depicted in Figures 3a and 3b, the dose distribution determined with the Gafchromic film in the 18 mm collimator, usually considered as reference field and calibrated with a 0.125 cm^3^ ionization chamber (7.25 mm length and 6.9 mm diameter) following the AAPM TG-21 protocol [8], is not completely homogeneous possibly due to the energy photon fluence variation. Although the absorbed dose distribution is more or less uniform in the centre of the field in the xy plane (axial plane relative to the patient position), the ionization chamber underestimates the dose due to the inhomogeneous area in the xz plane (coronal plane relative to the patient position) over which the dose is integrated. Such a result suggests that, even when agreement is observed between output factors calculated through Monte Carlo simulation or measured with different dosimeters, this does not warranty that the absorbed dose in the radiation field is properly known. Thus, one can argue that it is fundamental to be aware that not only the relative parameters are important, but the investigation of the reference dosimetry should also be an important matter.

**Table 4:** Output factors determined in the Leksell Gamma Knife® unit compared with that provided by the vendor.

		Collimator diameters (mm)			
Dosimeter	Size	4	8	14	18
MD-V2-55	∼240^a^	0.866±0.019	0.954±0.021	0.983±0.021	1.000
TLD-100	3.1×3.1×0.89^b^	0.839±0.029	0.948±0.043	0.966±0.038	1.000
Alanine	4.9^c^×3.0^d^		0.963±0.018	0.981±0.015	1.000
LGK data		0.868	0.955	0.984	1.000

a µm thickness,

b mm^3^,

c mm diameter,

d mm thickness.

For the data depicted in Table 3, it can be seen that within measurement uncertainties, there is good agreement between the Gafchromic film and TLDs, independent of the radiation field diameters equal to or larger than 10 mm. Interestingly, for the 35 mm diameter field, the absorbed dose to water rates computed with all three dosimeters statistically agree within 0.52%. It can be observed in Figure 4b that the ratio of the absorbed dose to water rate computed with ionization chamber following CD protocol and that determined with the Gafchromic film in the modified linear accelerator increases as the field size increases. This could be attributed to the lack of charged particles equilibrium as the field size decreases. Furthermore, the absorbed dose to water rate obtained through the conventional dosimetry for the 7.5 mm diameter field differs up to 11.6% with that determined with the Gafchromic film directly in the water phantom. This discrepancy might possibly be associated to the incorrect size, lack of spatial resolution and no-water-tissue-equivalence of the dosimeter used to measure the output factor in the accelerator under similar conditions [9]–[10]. Such a result is supported by most of measurements reported in the literature where differences of more than 14% have been observed in the measured output factors using various detectors and Monte Carlo simulation for radiation fields smaller than 20 mm diameter [12].

From the data reported in Figures 4a for the LGK, the mean ratio of the absorbed dose to water rate for each detector relative to the Gafchromic film is roughly independent of the field size. Instead, data depicted in Figure 4b for the linac indicates that the ratio increases as the field size increases. Such a different trend can be explained as follows. In the LGK, the absorbed dose to water rate in each single radiation field was determined by the product of the dose rate determined with the 0.125 cm^3^ ionization chamber and the output factors provided by the vendor which were obtained by Monte Carlo simulation. Thus, the main uncertainty comes from the perturbation produced by the ionization chamber in the 18 mm diameter reference field. In the linear accelerator, the absorbed dose to water rate in each small field is the result of the absorbed dose to water rate measured in a reference 10×10 cm^2^ field and the output factors measured by users. As mentioned above, even though charged particle equilibrium exists in the reference field, the output factor measurements strongly depend on the dosimetric characteristic of the detector used and smaller is the field, stronger is this dependence.

The evaluation of the relative combined standard uncertainties computed from the absorbed dose to water rate measurements for all collimator diameters studied and shown in Figure 5 indicates that the alanine dosimeter is the most accurate with an average relative combined standard uncertainty of (1.19±0.19)%, followed by the Gafchromic film with (1.41±0.12)%, and TLD with (2.36±0.67)%. Nonetheless, in spite of the high accuracy level provided by the alanine dosimeters in the direct measurement of the absorbed dose to water rate, they cannot be used in radiation fields smaller than 20 mm diameter due to their size. TLDs, besides being less accurate, also are not appropriate to measure absorbed dose to water rate in radiation fields smaller than 10 mm diameter. Finally, if a strict dosimetry protocol is followed, the Gafchromic film associated with a document flatbed scanner can be considered as a suitable methodology for reference dosimetry of small radiation fields used in stereotactic radiosurgery treatment modalities with acceptable accuracy (relative expanded uncertainty less than 3% at a 95% confidence level, i.e. *k = 2*), besides the low cost of the system.

### Conclusions

Reference absorbed dose to water rates were determined in 4 (4 mm–18 mm) Leksell Gamma Knife collimators and 5 (7.5 mm to 35 mm) cylindrical collimators from a modified linear accelerator using Gafchromic film, thermoluminescent and alanine dosimeters to investigate the suitability of well calibrated passive dosimeters as reference in small radiation fields with acceptable accuracy (relative combined standard uncertainty less than 1.5%). We have found that Gafchromic film, besides being a water-equivalent detector in this energy interval and having a high spatial resolution, can provide the adequate accuracy required for the absorbed dose measurement in small fields where steep dose-rate gradients exist. Based on these results, one can suggest the Gafchromic film as a suitable dosimeter to measure absorbed dose to water rate directly in water for small radiation fields used in stereotactic radiosurgery.
